# An expression atlas of human primary cells: inference of gene function from coexpression networks

**DOI:** 10.1186/1471-2164-14-632

**Published:** 2013-09-20

**Authors:** Neil A Mabbott, J Kenneth Baillie, Helen Brown, Tom C Freeman, David A Hume

**Affiliations:** 1 The Roslin Institute and Royal (Dick) School of Veterinary Studies, The University of Edinburgh, Easter Bush, Midlothian, Edinburgh EH25 9RG, UK

**Keywords:** Clustering, Meta-analysis, Human, Primary cells, Dendritic cell, Macrophage, Microarray, Transcriptomics

## Abstract

**Background:**

The specialisation of mammalian cells in time and space requires genes associated with specific pathways and functions to be co-ordinately expressed. Here we have combined a large number of publically available microarray datasets derived from human primary cells and analysed large correlation graphs of these data.

**Results:**

Using the network analysis tool BioLayout *Express*^3D^ we identify robust co-associations of genes expressed in a wide variety of cell lineages. We discuss the biological significance of a number of these associations, in particular the coexpression of key transcription factors with the genes that they are likely to control.

**Conclusions:**

We consider the regulation of genes in human primary cells and specifically in the human mononuclear phagocyte system. Of particular note is the fact that these data do not support the identity of putative markers of antigen-presenting dendritic cells, nor classification of M1 and M2 activation states, a current subject of debate within immunological field. We have provided this data resource on the BioGPS web site (http://biogps.org/dataset/2429/primary-cell-atlas/) and on macrophages.com (http://www.macrophages.com/hu-cell-atlas).

## Background

Living systems depend upon the concerted actions of numerous genes in pathways and macromolecular complexes. Underpinning these systems are complex transcriptional networks that drive the expression of subsets of the coding capacity of the genome to achieve a specialised function. The set of genes required for any cellular function must share transcriptional regulation, so that their products are available in the correct place at the right time. The potential utility of this information for the identification of candidate genes in human genetics has been emphasised previously [1-5]. The completion of genome sequences, the advent of microarray technologies, and advances in bioinformatic tools have revolutionised the ability to generate and analyse coexpression matrices. When translated into coexpression networks the information content of such networks depends to large extent upon the size and diversity of biological states sampled; the more states that are sampled, the more stringently one can state that a pair of genes share strict coexpression. Since, the pioneering efforts of Su *et al*. [6,7] to generate the Symatlas (now BioGPS, http://www.biogps.org) from data sets of microarray data from mouse and human tissues, there has been an explosion of gene expression “atlases” across multiple tissues and within tissues across cell types and developmental time ( [8-16]; http://www.immgen.org; http://www.brain-map.org; http://www.gudmap.org). These resources were recently extended by us to the domestic pig [17], a species of commercial importance for food production and a model in medical research [18].

Most major journals now require that array data is deposited in public depositories such as NCBI Gene Expression Omnibus and ArrayExpress. The availability of the primary data as well as the stabilisation of analysis platforms and methodology, permits meta-analysis of data produced in different laboratories [19]. The challenge then is to generate useful information from the microarray data deluge. The online meta-analysis dataset at EBI (http://www.ebi.ac.uk/gxa/) is a useful index for finding state-specific expression patterns, but does not readily provide a mechanism for finding genes with similar pattern. There are numerous different methods available for identifying clusters or modules of coexpressed genes within expression data [20-25]. Each could be argued to have its own advantages and a detailed review of these methods will not be included here. The method used in this study employed the network tool BioLayout *Express*^3D^ which was specifically developed to allow the visualisation and analysis of coexpression relationships in large datasets [26,27]. The tool identifies coregulated genes based on the construction of correlation networks, where genes (probesets) are represented as nodes, and edges represent the similarity (above a given threshold) between the expression profiles. Modules or clusters are then defined using the Markov Clustering algorithm (MCL) and both the network and clusters visualised using a powerful 3D network rendering engine. We have used this tool to identify coregulated genes in the mouse BioGPS dataset [2], and subsequently carried out a meta-analysis of available mouse data relating to hematopoietic differentiation [19]. Benita *et al*. [28] carried out a similar meta-analysis of human microarray data, with intention of identifying genes that were enriched in T cells relative to other cell types. Their rationale was that such enrichment would emerge from reference to a large number of immune and non-immune cells and tissues relative to T cells. The difficulty with including whole tissues in co-expression networks is that they are mixtures of cell types, including cells of the immune system. Conversely, the exclusion of tissues removes genes that are only expressed in mature, fully-differentiated cells *in vivo.* Furthermore, as noted by Benita *et al*. [28] static networks fail to sample inducible genes and the function of such genes may emerge from coexpression over time and in response to many different environments. An additional complication in human data, by contrast to inbred mice, is the impact of genetic variation. For example, Goring *et al*. [29] demonstrated that there was significant heritable variation in expression of a large proportion of transcripts detected using microarrays of peripheral blood. Robust coexpression analysis depends upon sampling many different datasets. In the present study, we present a meta-analysis of a large collection of microarray profiles of human primary cells available in the public domain.

## Results and discussion

### Source of expression data and method of analysis and quality control

**Figure 1:**
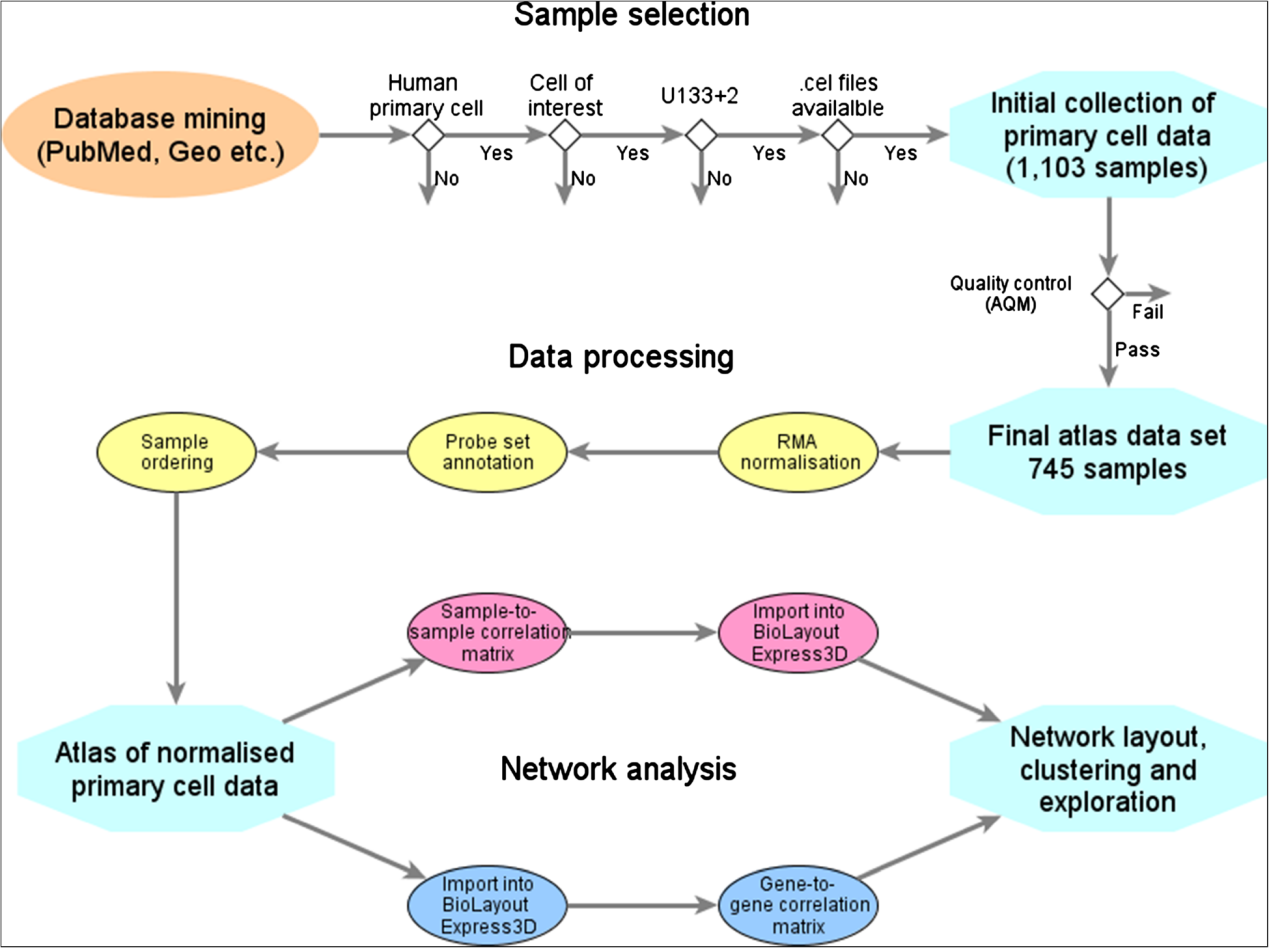
**Data analysis workflow.** Data analysis pipeline, from the selection of microarray data, through to normalisation, annotation and network analysis.

A large and diverse set of human primary cell gene expression data was collected, with a particular emphasis on datasets that divided immune cells into sub-populations based upon surface markers. Data sets were selected based on the following criteria: (1) chip platform (only data from Affymetrix Human Genome U133 Plus 2.0 expression arrays was included); (2) primary cell; (3) cell-subset studied; (4) availability of raw data (.cel) and sample description files. Quality control (QC) of these data using the arrayQualityMetrics package in Bioconductor showed a number of chips/data sets to be of poor quality or not comparable based on the chip signal intensities. Furthermore, additional datasets were rejected from this analysis following construction of initial network graphs, as these data showed inexplicable differences in their global expression intensities when compared to data from supposedly similar cells. Out of the 1,103 chips originally selected from 105 separate studies meeting the above criteria, 745 arrays passed the criteria for further analysis on the basis of these QC arrays. Additional file 1: Table S1 shows the range of cell populations represented in the remaining samples used for this analysis and their source. This table also provides additional information on the individual chip ID, cell class, stimulus or culture conditions; data series ID, individual chip ID and the Pubmed ID of the original study, if available. Samples were given a standard annotation (data series ID: cell class: chip description: replicate no.; Additional file 1: Table S1) and ordered by cell type (embryonic stem cells, induced pluripotent stem (iPS) cells, epithelial cells, fibroblasts etc.). The data analysis pipeline used in this study is shown in Figure 1 and the quality controlled normalised data used for this study is available from GEO: GSE49910.

### Clustering of co-expressed genes in a large collection of human primary cell types

**Figure 2:**
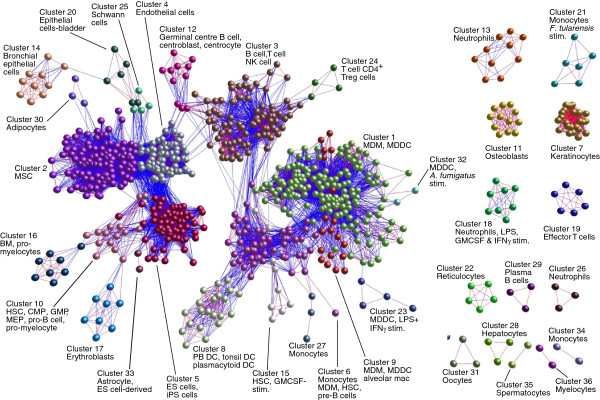
**Clustering of samples based upon their gene global expression profiles.** A Pearson correlation matrix was prepared comparing the data derived from all samples used in this study. A graph was then constructed using only those sample-to-sample relationships where *r* ≥ 0.9. Nodes represent samples and edges are coloured on a sliding scale according to the strength of the correlation (red, r = 1.0; blue, r = 0.9). The graph was then clustered using an MCL inflation value of 2.2, each cluster of samples being assigned a different colour. It is quite striking that almost without exception related cell types cluster together or are positioned within similar network neighbourhoods irrespective of the source of the data. Full details of the sources of all data sets used in the analysis is provided in Additional file 1: Table S1. Additional abbreviations used: CMP, common myeloid progenitors; GMP, granulocyte monocyte progenitors; HSC, haematopoietic stem cell; mac., macrophage; MDDC, monocyte-derived DC; MDM, monocyte-derived macrophage; MEP, megakaryocyte–erythroid progenitor cell; MSC, mesenchymal stem cells; NK, natural killer; PB, peripheral blood; stim., stimulated; Treg, regulatory T cell.

In order to compare global gene expression profiles and examine the relationships between these data as a whole we calculated a sample-to-sample Pearson correlation matrix on these normalised data. This matrix was then used to draw a graph of the sample-to-sample correlations using relationships *r* ≥ 0.9 to define edges (Figure 2). This graph shows a remarkable and reassuring consistency in the relationships between samples derived from similar cell types regardless of the laboratory from which they were generated. The different progenitor, myeloid, lymphoid and non-haematopoietic profiles clearly clustered like-with-like.

### Network analysis of the human cellular transcriptome

**Figure 3:**
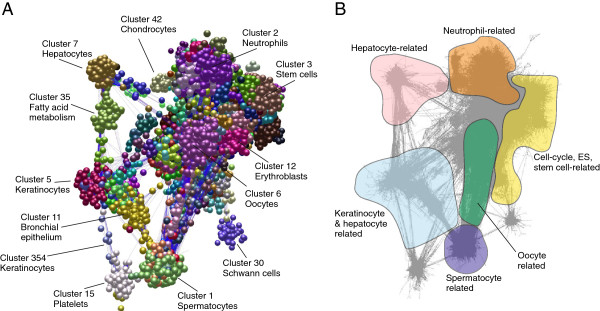
**Network analysis of human primary cell transcriptomics data. (A)** Main component of the network graph derived from 745 samples of human primary cell populations run on Affymetrix U133plus2.0 arrays. Nodes represent transcripts (probesets), edges represent correlations between individual expression profiles above *r* ≥ 0.75 and the colour of the nodes represents the cluster to which they have been assigned. The graph comprises of 24,808 nodes connected by 1,476,632 edges. **(B)** An image of the network graph showing edges only. Areas of enrichment of genes expressed in particular cell lineages are indicated.

A full probeset-to-probeset Pearson correlation matrix was then calculated using the tool Biolayout *Express*^3D^, whereby the similarity in the expression profile of each gene (probe set) represented on the array was compared across the 745 data sets. A network graph was constructed using a correlation threshold of *r* ≥ 0.75, whereby nodes represent individual Affymetrix probe sets and correlations between profiles greater than the selected threshold were represented by graph edges. The graph comprised 24,808 nodes connected by 1,476,632 edges and was subsequently clustered using the Markov clustering algorithm (MCL) at an inflation value (which controls the granularity of clustering) of 2.2. This resulted in 378 clusters containing more than 6 nodes. Transcripts in clusters smaller than this number were not assigned a cluster number. An image of the network graph is shown in Figure 3 with annotation of clusters of interest highlighted in distinct colours. The entire dataset is available on http://www.macrophages.com/hu-cell-atlas, where a webstart version of BioLayout *Express*^3D^ enables visualisation of the average expression of each cluster, and the specific expression of individual genes across the dataset. Additional file 2: Table S2 provides details of the probe sets within the entire set of 378 clusters as a Microsoft Excel worksheet. For comparison to whole tissue expression profiles, the table also includes a description of the clustering of the BioGPS human atlas dataset. To enable a convenient, user-friendly access to the data, we have also established a gene profile viewer for the data, reduced to show averaged expression levels for replicates on http://www.biogps.org. On this site, it is also possible to carry out a simple correlation search to identify genes with similar expression profiles to any selected gene of interest across the dataset.

**Figure 4:**
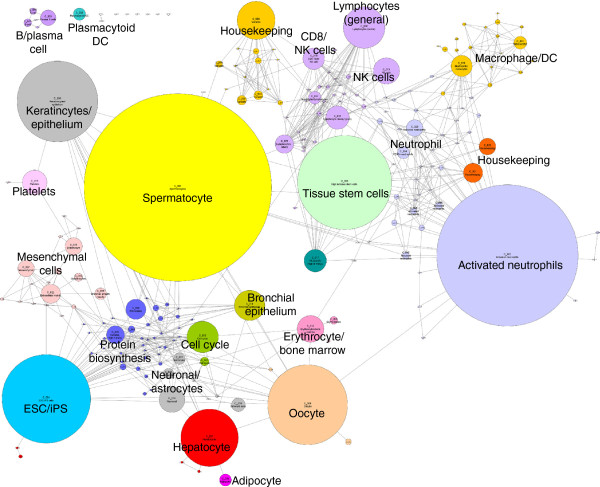
**Collapsed cluster diagram showing the relationship between cluster size and position within the network graph.** All the clusters derived from the network graph (Figure 3) with >10 nodes were “collapsed” such that each cluster is presented as a single node with size of the node proportional to the number of probe sets in the cluster. Edges represent instances where nodes in one cluster share correlations (*r* ≥ 0.75) with nodes in an adjacent cluster. Each cluster number is annotated with its number (C_00N) and clusters are coloured to reflect the underlying biological function or process which they are considered to represent.

The network graph derived from these data is large and its topology is complex. The graph’s obvious structure is derived from the grouping of genes which are expressed in a specific manner i.e. a correlation in their expression profiles of >0.75 and are therefore connected by a large number of edges forming cliques within the network. Some of these clusters represent genes expressed in a cell-specific manner, others not. The major structure of the graph is made up of a relatively small number (*n* = 50) of large clusters (>50 nodes), however, the majority (*n* = 328) are smaller in size. Another way of looking at such large transcriptional networks is to generate a collapsed cluster diagram. Figure 4 shows the relationship between cluster size and position of the cluster within the network. As a consequence, clusters comprising genes with a similar expression pattern tend to be in similar network neighbourhoods.

### Statistical assessment of the chances of these probeset-to-probeset correlations occurring by chance

To assess whether the probeset-to-probeset Pearson correlations we obtained using *r* ≥0.75 were important, one million simulated correlations were calculated from ‘pseudo-probesets’ generated at random. These ‘pseudo-probesets’ included 743 samples to match the number used in the original dataset above. Each ‘pseudo-probeset’ was generated by randomly selecting one of the original observed probeset values for each of the original 743 samples. Use of the original data to generate the ‘pseudo-probesets’ ensured that the underlying (non-normal) distribution of these data was reflected. Our analysis showed that only 0.0019% of the simulated correlations in these randomly generated data were above 0.75 (Additional file 3: Figure S1). Furthermore, whereas 0.32% of the observed correlations were above 0.75, only 0.000019% would be expected to have occurred by chance. These data clearly indicate that the probability of the probeset-to-probeset correlations at the level used in this study (*r* ≥0.75) occurring by chance was very small.

### Functional annotation of genes involved in generic pathways and processes

The average expression profiles of the genes in the largest 50 clusters are available in Additional file 4: Figure S2. As we noted previously in an analysis of the mouse BioGPS data, many of the clusters are not cell lineage restricted, but rather reflect variation in the activity of generic metabolic functions amongst the cells. For example **Cluster 20** is enriched in ribosomal genes (gene ontology (GO) term 0003735, structural constituent of ribosome, corrected *P* value 2.01×10^-130^) which are expressed highly by almost all cell populations. These genes form a distinct cluster because of the relative absence of expression from neutrophils, consistent with their known relative low rates of active protein synthesis and the acute regulation of translation during myeloid differentiation [30]. Also situated in the same neighbourhood of the graph and connected to **Cluster 20** by a number of edges is **Cluster 78** which contains a small set genes encoding proteins involved with RNA splicing and the spliceosome (GO:0003723, RNA binding, *P* < 8.28×10^-12^; GO:0005681, spliceosome, *P* < 6.47×10^-5^). Almost all of the genes in **cluster 78** have been implicated in alternative splicing, e.g. recent studies of *KHDRBS1*, aka Sam68 [31]. By contrast, the large majority of the other splicing factors are not part of significant clusters. This suggests the existence of specifically-regulated alternative splicing complexes with idiosyncratic regulation [31]. **Clusters 10** and **41** are highly enriched in genes associated with the cell-cycle and mitosis and are expressed at high levels by proliferating cell-types such as embryonic stem cells, iPS cells and bone marrow (BM) progenitor cells (**cluster 10**, GO:0007049, cell cycle, *P* = 0; **cluster 41**, GO:0007049, cell cycle, *P* = 7.57×10^-7^). Almost all of the genes in these clusters have known functions that are evident from their names, in cycle control, DNA synthesis, DNA repair, mitotic spindle formation etc. Some genes with uninformative names (according to Affymetrix annotation) such as *KIAA0101* actually have a known function in S phase [32]. Others, such as *FAM83B* and *HMMR* are amongst many genes identified via a high throughout screen to identify proteins that control mitosis (http://www.mitocheck.org). A recent study by Tipton *et al*. [33] used data mining to identify 64 genes that are core components of the kinetochore complex, and then sought evidence of other members of the complex based upon coexpression analysis and/or protein-protein interactions in public domain data. **Clusters 10** and **41** contain all of the 64 core kinetochore genes identified by these authors, as well as the novel gene, *TRIP13*, that they identified and validated. These clusters also contain 41/50 of the top 50 candidate interacting genes, and around 100 additional genes, the large majority of which are obviously involved in the cell cycle. We therefore reannotated all of the Affymetrix probes in these clusters. In keeping with the concept of guilt-by-association, there is published evidence (PMID citations included in Additional file 5: Table S3) of likely cell cycle roles for the large majority of genes with uninformative annotations and no associated GO terms (Additional file 5: Table S3). The interesting feature of the two clusters is that **Cluster 10** contains the well-known transcriptional driver of mitosis, FOXM1 [34] as well as the E2F family repressors, E2F7 and E2F8. Other members of the proliferation-associated E2F family, including E2F1, are not within the cluster despite the fact that many of the genes are known E2F1 targets. Indeed E2F1 and E2F4 are not themselves within the annotated cell cycle clusters but are within the neighbourhood (not shown). E2F1 is clearly regulated during the cell cycle, driving the entry into S phase, but its expression level declines, as its target genes increase in expression. E2F1 functions are also regulated post-transcriptionally via interactions with Rb [35]. **Cluster 41** does not contain an obvious transcriptional regulator, but could contain downstream targets of **Cluster 10**.

### Identification of clusters containing cell-specific transcriptomes

Cells of mesenchymal origin all expressed genes in **Clusters 22, 25, 27, 36, 42** and **70** at high levels; these were highly enriched in genes encoding components of the extra-cellular matrix shared by the different mesenchymal lineages (GO:0005578, proteinaceous extracellular matrix, Additional file 2: Table S2), as previously identified in mouse data [2,5]. The clusters are segregated because of relative enrichment in the different mesenchymal cell types; the expression of genes in **Cluster 22** is relatively higher in osteoblasts and contains many genes with “osteoblast” in their name, but clearly not entirely osteoblast-restricted. The genes within this cluster are also expressed in other mesenchymal cell types (fibroblasts, tissue stem cells, chondrocytes), but notably not endothelial cells and overlap the extracellular matrix clusters we have described previously in mouse datasets [2,5,19].

Genes in **Cluster 91** were widely expressed in samples other than embryonic stem (ES) cells, iPS cells and gametocytes, and almost exclusively encoded proteins associated with the major histocompatibility complex (MHC) class I (GO:0002474, antigen processing and presentation via MHC class I, *P* < 1.06×10^-16^). In contrast, **Cluster 89** contained genes encoding MHC class II proteins, expression of which was mostly restricted to BM progenitors, monocytes, mononuclear phagocytes and B cells (GO:0042613, MHC class II protein complex, *P* < 8.72×10^-16^). Apart from the MHC (human leukocyte antigen, HLA) genes, the cluster contains the invariant chain *CD74*, *LY86* and *CST3* (cystatin C) all known to be involved in antigen presentation. Unexpectedly, it also contains adenosine deaminase II gene, *CECR2*, and the signalling molecule *CARD9*, which could have a role in antigen presentation [36].

In addition to the identification of a number of clusters shared by groups of cells that appeared to be related to specific functions or processes, a large number of clusters had expression restricted to, or greatly enriched in, individual cell populations. The largest of all, **Cluster 1**, is expressed almost exclusively in spermatocytes, contains obvious index genes such as acrosomal vesicle protein 1 (*AVCR1*) and is enriched in genes involved in spermatogenesis (GO:0007283, spermatogenesis, *P* < 2.9×10^-59^).

### Identification of putative cell-specific transcription factors

A full description of the expression profile of the top 100 clusters is provided in Additional file 2: Table S2. A highlight of each of these clusters is the presence within them of likely lineage-restricted transcription factors. A good example is the second largest cell-type enriched cluster, **Cluster 4**, which contains genes expressed specifically in ES cells and iPS cells. There is a substantial overlap with the ES cell-specific cluster identified previous from the mouse BioGPS data [2]. The cluster includes the known ES cell-associated transcription factors and pluripotency markers; *LIN28A, NANOG, GLI2, POU5F1 (Oct4), ROR1, SALL2, SALL3, SALL4, SOX2, TCF7L1, ZIC2, ZIC3 and ZIC5.* Surprisingly, it also contains the germ cell-associated genes *SRY* and *PRDM14*, and transcription factors associated with lineage commitment such as *FOXA3* and *FOXH1* suggesting that many of the cultures included in the data set are partly-differentiated. The GO term analysis confirmed this cluster was significantly enriched with genes involved with transcription (GO:0005634, nucleus, *P* < 3.53×10^-29^; GO:0006350, transcription, *P* < 5.26×10^-9^).

**Cluster 30** contains the transcription factor *FOXD3*, which is implicated in neural crest lineage determination [37], desert hedgehog (*DHH*) and its target *SOX10*, which are implicated in Schwann cell formation [38] and *RXRG*, which is implicated in control of remyelination [39]. Expression of this cluster was restricted to Schwann cells and contained many genes related to myelin formation and neuronal transmission (GO:0007278, synaptic transmission, *P* < 0.00793; GO:0007399, nervous system development, *P* < 0.00793) including myelin basic protein (*MBP*), myelin protein zero (*MPZ*), dystroglycan 1 (*DAG1*) and proteolipid protein 1 (*PLP1*). The cluster contains a significant number of unannotated probes and hypothetical proteins. One of them, *MGC45800*, is amongst the genes associated with susceptibility to multiple sclerosis [40].

The transcription factor *ERG,* implicated in endothelial differentiation [41], and the related factors *SOX7, SOX17* and *SOX18,* which have partly redundant functions in angiogenesis [42] are contained with **Cluster 25** (GO:0001525, angiogenesis, *P* < 2.46×10^-5^). The genes within this cluster are expressed at highest levels by endothelial cells and contained many encoding endothelial growth factor receptors (*ACVRL1, KDR, TEK, TIE1, PROCR*), adhesion molecules (*ESAM*, *ICAM2*, *MCAM*) or components of the extracellular matrix such as heparan sulphate proteoglycans (*GLCE*, *HSPG2, ST6GALNAC3)*. Interestingly, a much smaller cluster, **Cluster 196**, also enriched in endothelial cells, contains *ANGPT2, HOXD1, FLT4* and *SPRY1,* each with known endothelial-specific biologies. This implies that these genes interact specifically in some aspect of endothelial differentiation, and also strongly implicate the poorly-annotated gene *TM4SF18.***Cluster 42** (GO:0005578, proteinaceous extracellular matrix, *P* < 6.04×10^-18^; GO:0001501, skeletal development, *P* < 1.58×10^-17^) contains the chondrocyte differentiation factor, *SP7*[43]*,* as well as *NKX3-2* and its regulator Indian Hedgehog (*IHH*) [44] and the many known chondrocyte-specific markers (e.g.: *CLIP2, CHAD*) and cartilage collagens which distinguish these cells from osteoblasts, which express genes especially enriched in **Cluster 22.**

**Cluster 12** contains the erythropoietin receptor, *EPOR,* and the key red cell transcription factors, *KLF1*, *TAL1, GATA1, GFI1B* and *SOX6*[45]. Genes within this cluster were expressed at high levels by erythroblasts and include the globins, many red cell structural proteins and key members of the heme biosynthesis pathway *ALAS2*, *ALAD*, *HMBS*, *UROS*, *UROD*, *CPOX*, *PPOX* and *FECH* (GO:0006783, heme biosynthetic process, *P* < 9.92×10^-13^)*.***Cluster 7** contains the liver-specific transcription factors *FOXA2, NROB2, NR1H4* (the bile acid receptor), *NR1I2, NR1I3, HNF4A* and *HNF4G,* and most of the genes within it are related to liver function and expressed at highest levels by hepatocytes. These included albumin (*ALB*), members of the cytochrome P450 family, transferrin (*TF*), most blood coagulation cascade components, complement components and related factors (eg: *C3P1, C4BPA, C4BPB, C5, C6, C8A, C8B, C8G, C9, CFHR2, CFHR3, CFHR4, CFHR5, CFI, MASP2* and *MBL2*), coagulation factors (eg: *F2, F7, F9, F10, F11, F12* and *F13B*), and many genes involved in alcohol (*ADH1A, ADH1C, ADH4, ADH6)*, carbohydrate and lipid metabolism (GO:0006629, lipid metabolic process, *P* < 2.36×10^-36^; GO:0016491, oxidoreductase activity, *P* < 4.24×10^-34^).

### Comparison of the transcriptomes of specific human immune cell subsets

As seen in our previous meta-analysis of mouse microarray data, the different leukocyte lineages can be defined by sets of coregulated genes. The largest leukocyte-enriched cluster is **Cluster 2,** a set of genes almost exclusively expressed in neutrophils purified using a novel isolation methodology [46]. This involved an affinity capture of the cells using anti-CD66B antibodies, and stimulation with either bacterial lipopolysaccharide (LPS), or granulocyte-macrophage colony-stimulating factor (GM-CSF) plus interferon (IFNγ). Curiously, this cluster does not contain many of the known granulocyte markers, but instead overlaps significantly with genes identified as “housekeeping” in our previous analyses in mice [2] (GO:0006350, transcription, *P* < 3.77×10^-13^; GO:0019222, regulation of metabolic process, *P* < 4.14×10^-13^; GO:0005634, nucleus, *P* < 4.99×10^-12^). It contains a number of transcription factors generally regarded as ubiquitous and/or involved in cytoprotective pathways, notably *ATF5* and *ATF7* and *SP1, 2 and 3,* but also contains some evidence of JAK-STAT pathway activation evidenced by the presence of *STAT5A* and the feedback regulators *SOCS1* and *SOCS4*. The cluster also includes the RNA polymerases, RNApol II and RNAPol III and subunits *TAF13* and *TAF15*. Neutrophils are known to have relatively low levels of RNA and protein synthesis. We suggest that being placed in culture has initiated a relatively synchronous induction of generic biosynthetic pathways in these isolated neutrophils. Within the cluster, there are also lineage-related transcription factors, notably *E2F3* (which has a role in myeloid differentiation; [47]) and *RUNX1*, *CEPD*, and ETS family proteins *ETS2*, *ETS3*, *ETS5* and *ETS7*. Although the inclusion of these neutrophil datasets in some measure distorts the analysis, for the purpose of genome annotation and guilt-by-association it clearly associates many genes with associated functions and it contains many genes with uninformative gene names or lacking annotation. A separate, much smaller, **Cluster 19,** is enriched in uncultured neutrophils and BM, and contains the granulocyte colony-stimulating factor (G-CSF) receptor (*CSF3R*) and the transcription factor *STAT5B*. Other leukocyte clusters tend to be rather smaller than one might anticipate from stereotypical views of “lineages”, and are more associated with known functional pathways. **Cluster 16** contains the CD3 components, the T cell receptors, and the signalling factors *ZAP70, LCK, JAK3 and PKCQ* and is enriched in T cells (GO:0042110, T cell activation, *P* < 2.14×10-8; GO:0042101, T cell receptor complex, *P* < 4.16×10^-7^). The cluster also contains the gene encoding FLT3-ligand (*FLT3LG*), the major inducer of antigen-presenting cell differentiation [48]. The transcription factor within this cluster is LEF1, also known as T cell factor [49]. **Cluster 29** is the corresponding plasma cell/ B cell-specific cluster (GO:0003823, antigen binding, *P* < 2.04×10^-5^), containing the immunoglobulin genes, and B cell maturation antigen, *TNFRSF17*. The presence of the little studied transcription factor, PRDM15, in this cluster may infer a function in B cell differentiation.

**Clusters 31** (GO:0005764, lysosome, *P* < 1.63x10^-10^) and related **Cluster 123** (GO:0006812, cation transport, *P* < 0.00205) contain the genes previously identified in mice and humans as being associated with phagocyte function including *LAMP1*, vacuolar ATPase subunits and lysosomal hydrolases, and the transcription factors that regulate them, notably *CEBPA* and *MITF*[2]. **Cluster 33** is enriched in plasmacytoid dendritic cells (pDC) and contains *TLR9,* known to be pDC-restricted in humans, and the pDC transcription factors *IRF4* and *SPIB*[50], again consistent with previous mouse data [2]. It is not our purpose to annotate all of the clusters processively. As we progress to smaller regulons we see rather tight associations of genes that have obviously-related functions. For example, **Cluster 62** is enriched in monocyte-derived macrophages and dendritic cells (DC) and contains a set of genes that may have a function in internalisation of apoptotic cells. **Cluster 66** contains many genes involved in cytotoxic T cell and NK cell cytotoxic activity, including perforins and granzymes (GO:0001906, cell killing, *P* < 0.000178). The neighbouring **Clusters 88** and **99** (GO:0009615, response to virus; *P* < 2.58×10^-8^ &*P* < 0.00978, respectively*)* contain distinct sets of the known antiviral effectors amongst type 1 IFN target genes including: *DDX60*, *MX1*, *MX2, OAS1*, *OAS2*, *OAS3;* and *DDX58*, *IFIT1, IFIT2, IFIT3*, *IFIT5,* respectively. This suggests that there is subtle discordance in the regulation of the IFN response. **Cluster 93** contains a small subset of the known Toll-like receptor (TLR)-inducible cytokines and chemokines including *CXCL’s 1,2,3* and *IL1B* and *IL8* (GO:0008009, chemokine activity, *P* < 5.4×10^-10^).

As noted in our previous meta-analysis of large mouse microarray datasets [19], the maturity of cDNA microarray technologies has made it possible to compare profiles based upon their biology and not according to the laboratory that generated them, a particular problem in the early days of microarray analyses [51]. As demonstrated in our meta-analysis (Figure 2), with modern microarrays, datasets from the same cell type but generated in different laboratories can be remarkably similar. For example, although endothelial cells from five independent sources were included in this analysis (Additional file 1: Table S1) each data set was contained within the same cluster of the sample-to-sample correlation graph (Figure 2, **cluster 4**).

Part of the purpose of the current analysis was to generate a microarray resource to be displayed on the website http://www.biogps.org, to complement the current tissue data set that was assembled in 2004, and which is still shown as a resource on several genome browsers. The current human dataset on BioGPS has very limited representation of primary cells, especially those of the immune system. In our previous analysis of the mouse BioGPS data, we demonstrated the greater information content that could be derived from a focus on purified cells, as opposed to tissues [2,19]. To demonstrate the utility of the human cell metadata, we have carried out a clustering analysis using the network tool BioLayout *Express*^3D^. The detailed analysis of the genes involved in the cell cycle, which clearly differ across this very large data set depending upon the proportion of cells actively involved in cell proliferation, provides a further clear indication of the power of “guilt-by-association” in the annotation of the likely function of genes with uninformative gene names. In the same manner, from a gene expression atlas for the domestic pig, we extracted very clear coexpression of the genes involved in oxidative phosphorylation [17]. The recent assertion by Gillis and Pavlidis [52] that guilt-by-association is the exception rather than the rule in gene networks is clearly incorrect in mammalian systems. Their argument is based in part upon the nature of networks; gene products that have numerous interaction partners (high node-degree) tend to be involved in any process you care to look at. The example that they use is *TP53*, the well-studied tumour suppressor gene encoding p53. Perhaps because it has so many functions and partners, *TP53* is actually idiosyncratic in its regulation and the two probes designed to this gene lie in an small isolated graph component consisting of 3 transcripts (together with YWHAE a protein known to associated with P53). By contrast, the related *TP73* gene is strongly enriched in bronchial epithelium (**Cluster 11**) and has been ascribed roles as a tumour suppressor in the lung [53]. More importantly, their analysis is based largely upon the limited perspective of yeast and/or the still limited information content of GO terms. As we have also shown previously in studies of the mouse, the principle of guilt-by-association works well when one analyses very large datasets of different cell types from a mammal. Because individual cells have specialised functions, the gene products required for those functions must be present in the same cell at the same time, and the underlying regulation is predominantly via control of transcription. Furthermore, the importance of such coexpression information is evident in analysis of genetic data. In simple terms, one can infer the likely phenotype of a mutation in any specific gene from its pattern of expression [1,4]. For the purpose of the current analysis we chose a threshold for the network graph of 0.75. Less stringent correlations may still be informative. Using the “correlation” tool on the BioGPS website, one can find the closest “friends” of any gene on the arrays. For example, *IFITM2* and *IFITM3*, which are neighbouring IFN-induced genes involved in intrinsic antiviral defence, probably arose from gene duplication and which have highly-conserved promoters, are correlated at around 0.7 across this large data set. An even higher correlation is seen using this tool on the mouse BioGPS data, which may partly reflect the fact that these are inbred animals. Conversely, large clusters such as **Cluster 2,** which contains a mixture of known myeloid/granulocyte-enriched genes and general anabolic genes, may reveal greater information content if a higher threshold is chosen. The function of genes within **Cluster 2** would also be more thoroughly dissected if granulocyte lineage cells were more thoroughly polled, but quality datasets for this purpose were not available. The datasets chosen for this analysis were also focussed in part on strong datasets from cells of the monocyte-macrophage lineage. As observed previously in analyses of mouse data, macrophages and DC (other than lymph node-derived and pDC) cluster together in terms of their overall profiles. Surface markers that have commonly been used to separate the cells show no evidence of association with other functions and many of them form no cluster at all. The two subunits *CD11B* (*ITGAM*) and *ITGB2*, are correlated with the focal adhesion protein *FERMT3* and *LSP1*, in the very small **Cluster 169**, which further validates the approach. Class I MHC genes are within a single cluster, **Cluster 91** (GO:0002474, antigen processing and presentation via MHC class I, *P* < 1.06×10^-16^), and as one might expect, coregulated with beta2-microglobulin, but not with the antigen-processing genes *TAP1* and *TAP2* which appear independently-regulated. Rather less obvious is the association of the lysosome protein transmembrane 5 (*LPTM5*) with this cluster, which could suggest a function in antigen presentation. The Class II MHC genes are mainly within a single cluster, **Cluster 89** (GO:0042613, MHC class II protein complex, *P* < 8.72×10^-16^), and all of the unannotated probes within this cluster also map to MHCII but there is no association with any other marker or putative antigen uptake receptors such as LY75 (*DEC205, CLEC13B*). Hence, as in mouse, there is no surface marker that can be used to define antigen-presenting cells, or to distinguish macrophages from DC other than class II MHC *per se*. The costimulators, *CD83* and *CD86* also show no association with each other or with class II MHC (*HLADR, HLADP* and *HLADQ*).

We also chose to include multiple macrophage datasets in this analysis in part because they are amongst the most complex sources of mRNA, and can respond to numerous distinct stimuli with massive changes in gene expression [54]. We also aimed to determine whether there was a robust set of genes that can define the polarisation of macrophages towards M1 or M2 activation states [55]. Across this very large data set, there are sets of inducible genes that are robustly co-regulated, notably the two distinct sets of IFN target genes (**Clusters 88** and **99**) and the immediate early inflammatory genes including *IL1B* and *IL8* (**Cluster 93**). None of the genes proposed to distinguish the M1/M2 polarisation states in human monocyte-derived macrophages [56], including the surface markers such as *MSR1, MRC1, CD36, DCL1* and *CD209*, show any evidence of coregulation within a cluster. Furthermore, two of the most-studied proinflammatory cytokines, *IL6* and *TNF*, are also not included within any coexpression clusters. There are several reasons why genes that have been considered as markers for particular activation states in myeloid cells do not correlate well with each other if one examines much larger data sets. Firstly, the data we are examining come from many different outbred humans, rather than limited numbers of donors or inbred mice, and involves many different stimuli. There are well-studied promoter polymorphisms affecting the proinflammatory cytokines and their receptors. Indeed, the expression of as many as half of the genes detectable in leukocytes may be affected by cis-acting variation [29]. So, genetic variation amongst donors probably reduces the apparent correlation amongst induced genes, and such variation probably contributes to infectious and inflammatory disease susceptibility [57]. Secondly, the simple concept of dichotomy of polarisation states is probably wrong. Each gene has its own promoter and its own idiosyncratic response to a common transcription milieu [58,59], and each agonist acts upon different classes of receptors and therefore acts differently on the available inducible genes. So, the number of polarisation states is essentially infinite and the divisions are arbitrary and artificial. The other interesting feature highlighted by the network analysis is that many of the cell lineage-restricted clusters contain only one, or a very small number of, transcription factors. In all cases we have examined, those factors are well known to have non-redundant roles in lineage-restricted transcription and determination. Of course, no cell lineage is determined, nor a gene cluster regulated solely, by a single transcription factor. When we consider macrophage differentiation, there are multiple genes involved. PU.1 (*SPI1*) has been ascribed a central role, but most recent evidence indicates that its function is permissive, establishing a chromatin state that is subsequently available to other regulators [54]. The *SPI1* gene is actually not contained with the phagocyte cluster, because it is also expressed in neutrophils and B cells and has a unique pattern of regulation upon activation. There is an unexpected set of coregulated genes (**Cluster 72**), including *AIF1, LST1, LILRA1* and *LILRA2* that we might consider as candidate direct PU.1 targets. Phagocyte-specific genes have purine-rich promoters that bind PU.1 but based upon the co-expression analysis, we would suggest that PU.1 is necessary, but not sufficient, and *CEBPA* and *MITF* (or other MIT family members, *TFEC, TFE3* and *TFEB*) are the absolute determinants of expression. These two regulators probably regulate each other. The *CEBPA* promoter itself contains a conserved *MITF* recognition motif (CCAGCTG, E-Box) immediately upstream of the transcription start site (http://www.ensembl.org). *MITF* is expressed in both humans and mice from multiple promoters [60,61].

## Conclusions

In summary, we have generated a resource for functional annotation based upon the meta-analysis of gene expression data from human primary cells. All these data have been uploaded to the BioGPS website to provide a user-friendly resource enabling the identification of transcriptional friends of human genes. Our conclusions based upon the unbiased clustering of gene expression with BioLayout *Express*^3D^ contrast with recent studies on the mouse by the ImmGen consortium [62,63], in which the authors have sought to identify marker genes based upon a preconception of the separate identity of macrophages and DC. One of these studies reported a small set of genes which distinguished mouse cDC from four prototypical tissue macrophage populations [57]. Additional file 6: Figure S3 shows the average expression profiles of these proposed mouse core cDC signature genes [57] across all of the human myeloid cell data sets used in the current study. These data show that in humans such markers do not define either cell culture-derived or lymphoid tissue “DC” in an unbiased analysis of gene expression profiles. This conclusion is consistent with our own independent studies of the mouse [2,19] and reanalysis of the ImmGen data (http://www.macrophages.com/HumeNI2013, [64]). Antigen presentation and phagocytic activity are functions that require coordinated gene expression, they do not define cell types or lineages.

## Methods

### Selection of gene expression data sets

The NCBI Gene Expression Omnibus database (http://www.ncbi.nlm.nih.gov) was searched for human primary cell expression datasets. Data sets were selected based on the following three criteria: (1) chip platform (Affymetrix human genome U133 plus 2.0 expression arrays); (2) cell type studied; (3) availability of raw data (.cel) files. Accordingly, a diverse set of human leukocyte gene expression data was collected comprising a total of 1,103 chips from 105 separate studies. All raw data (.cel) files were downloaded and the quality of the raw data from each dataset was reanalysed using the arrayQualityMetrics package in Bioconductor (http://www.bioconductor.org) and scored on the basis of 5 metrics, namely maplot, spatial, boxplot, heatmap and rle. Any array failing on more than one QC metric was removed from the dataset. Normalisation of all data was performed independently using the robust multi-array average (RMA) expression measure [51]. Probesets were then annotated using latest annotation available in Bioconductor (26 June 2009) and samples ordered according to cell-type grouping to ease interpretation of the data (iPS cells, ES cells, BM, BM progenitors, macrophages, lymphocytes etc.).

### Network analysis

All normalised data passing the QC was saved as an ‘.expression’ file. This file contains a unique identifier for each row of data (Gene symbol concatenated to probeset ID), followed by columns of gene annotations which can be used as class-sets for the overlay and analysis of information with respect to the graph and finally natural scale normalised data values for each sample (each column of data being derived from a different sample). This file was first used to prepare a sample-to-sample correlation matrix using the ‘cor’ package with Bioconductor. This was imported into the tool BioLayout *Express*^3D^[26] and a graph plotted using all sample-to-sample relationships >0.9. Next, using BioLayout *Express*^3D^ a pairwise Pearson correlation matrix was calculated thereby performing an all vs. all comparison of the expression profile of each probeset on the array. All Pearson correlations where r ≥ 0.7 were saved to a ‘.pearson’ file. Based on the analysis of the initial network graphs additional datasets were rejected as they showed global differences in their expression profiles when compared to data from supposedly similar cell types. Out of the 1,103 chips originally selected, 745 arrays were selected for further analysis on the basis of these QC measures. Based on a user defined threshold of r = 0.75, an undirected network graph of the data was generated. In this context nodes represent individual probesets (genes/transcripts) and the edges between them Pearson correlation coefficients above the selected threshold. The resulting graph was large and highly structured. The network was then clustered into groups of genes sharing similar profiles using the MCL algorithm with an MCL inflation value (which controls the granularity of clustering) set to 2.2.

The graph of the combined datasets was explored extensively in order to understand the significance of the gene clusterings and the functional activity of the cell populations investigated. Genes in the clusters of interest were assessed for cellular function using a combination of literature review and bioinformatics. Significantly over-represented gene ontologies within clusters of interest were identified using GOstat (http://gostat.wehi.edu.au). For each GO term, the probability was calculated that the observed counts occurred by the random distribution of this GO term between the cluster of interest and the reference group (genes on the microarray). The Benjamini and Hochberg correction was used to control the false discovery rate of errors expected from multiple testing. Over-represented gene ontologies with *P* values < 0.05 were accepted as significant (Additional file 2: Table S2). Groups of genes often shared several GO terms that were indicative of the same biological process, molecular function or cellular compartment. In these instances the most informative GO terms within the top 10 identified are presented.

### Availability of supporting data

The entire dataset is available on http://www.macrophages.com/hu-cell-atlas, where a webstart version of BioLayout *Express*^3D^ enables visualisation of the average expression of each cluster, and the specific expression of individual genes across the dataset. All these data have also been uploaded to the BioGPS website (http://biogps.org/dataset/2429/primary-cell-atlas) to provide a user-friendly resource enabling the identification of transcriptional friends of human genes.

## Abbreviations

BM: Bone marrow; DC: Dendritic cell; ES: Embryonic stem; G-CSF: Granulocyte colony-stimulating factor; GM-CSF: Granulocyte-macrophage colony-stimulating factor; GO: Gene ontology; HLA: Human leukocyte antigen; IFN: Interferon; iPS cell: Induced pluripotent stem cell; LPS: Lipopolysaccharide; MCL: Markov clustering; MHC: Major histocompatibility complex; pDC: Plasmacytoid dendritic cell; QC: Quality control; RMA: Robust multi-array average; TLR: Toll-like receptor.

## Competing interests

The authors declare that they have no competing interests.

## Authors’ contributions

TCF, DAH and NAM conceived the study. JKB, HB, TCF, DAH and NAM performed the computational analysis and drafted the manuscript. All authors read and approved the final manuscript.

## Supplementary Material

Additional file 1: Table S1 A full description of the cellular identities and treatment conditions of each dataset included in the analysis and their order of presentation. The source of the data (GEO identifier) is also provided. Click here for file

Additional file 2: Table S2 An Microsoft Excel file detailing all of the coexpression clusters derived from this analysis including the gene/probeIDs within the cluster and cluster description annotation. Click here for file

Additional file 3: Figure S1 The probability of the probeset-to-probeset correlations at the level used in the current study (*r* ≥ 0.75) occurring by chance is very low. Histogram shows the distribution of the actual (blue bars, %) and randomly simulated probeset (red bars, %) chance correlations for a range of Pearson correlation values. The boxed area on the *x*-axis of the main histogram is shown in detail in the inset panel. Click here for file

Additional file 4: Figure S2 Average expression profile of all transcripts present in the 50 largest clusters from these analyses. *x*-axis displays the grouping of the different primary 590 cells analysed and the *y*-axis the average normalised expression signal of all transcripts in cluster. The cluster number is shown (C_00N), together with the annotated cluster name and the number of transcripts it contains (in brackets). Click here for file

Additional file 5: Table S3 An annotated description of the cell cycle-associated clusters 10 and 41. Click here for file

Additional file 6: Figure S3 Expression of mouse dendritic cell ‘marker’ genes across human myeloid cell types analysed in these studies. The list of genes shown here was recently published by Miller *et al.* (2012) as defining mouse dendritic cells based on the analysis of a subset of the ImmGen data [62,63]. We have suggested that this is not really the case in mice and this figure would suggest that this does not hold true in humans either. Horizontal colour bars represent the myeloid sub-types analysed, the histogram bars the mean 600 expression value for the replicates of an individual sample type from a study, number of samples averaged is shown in brackets. Click here for file
