# Supplementary figures and images for: An expression atlas of human primary cells: inference of gene function from coexpression networks
Source: BMC Genomics. 2013 Sep 20;14:632. doi: 10.1186/1471-2164-14-632 (PMC3849585; doi:10.1186/1471-2164-14-632)

**Figure S1:** Mabbott *et al.*

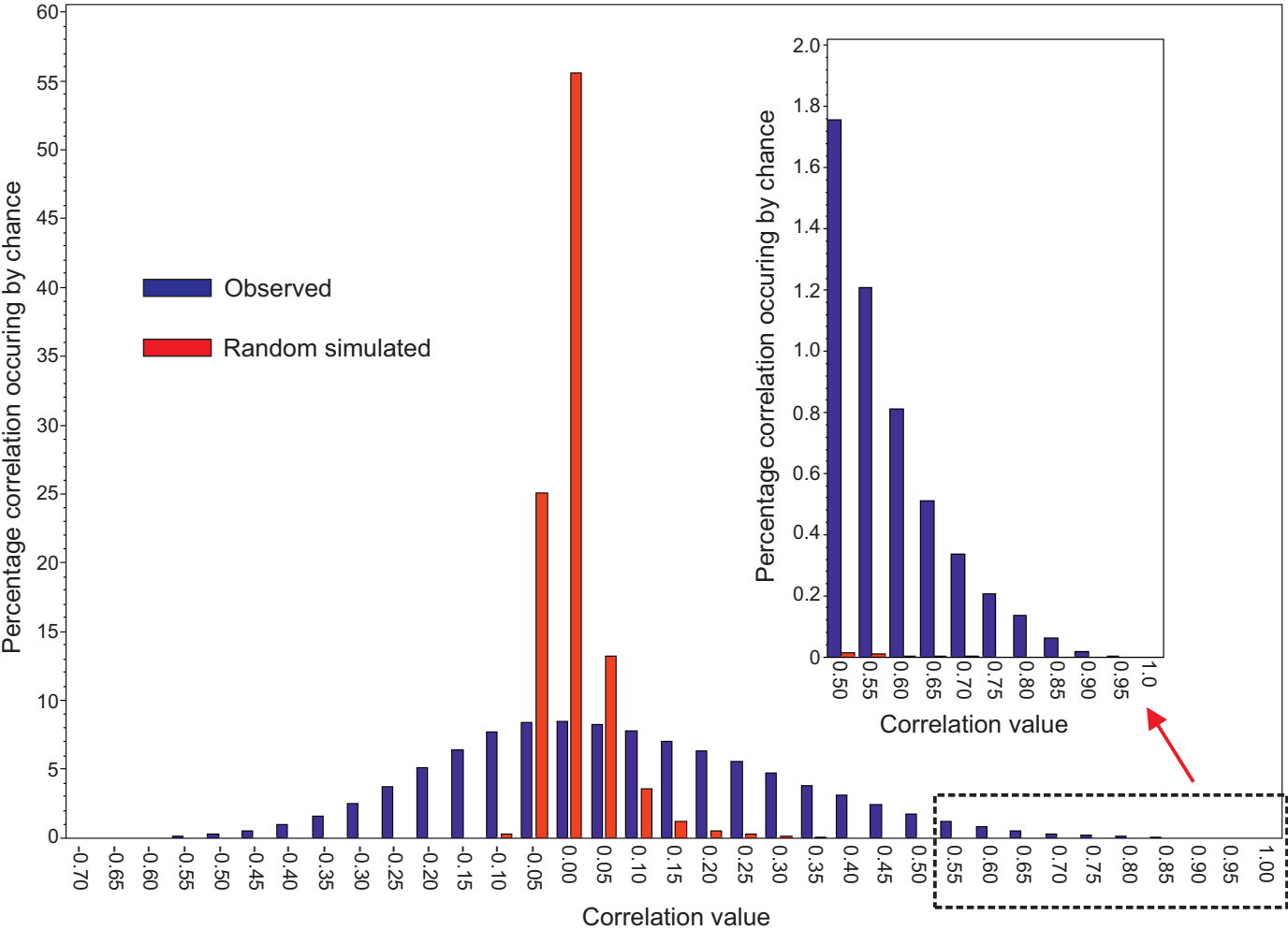

Supplement: Additional file 3: Figure S1 — The probability of the probeset-to-probeset correlations at the level used in the current study (r ≥ 0.75) occurring by chance is very low. Histogram shows the distribution of the actual (blue bars, %) and randomly simulated probeset (red bars, %) chance correlations for a range of Pearson correlation values. The boxed area on the x-axis of the main histogram is shown in detail in the inset panel. [file 1471-2164-14-632-S3.pdf]

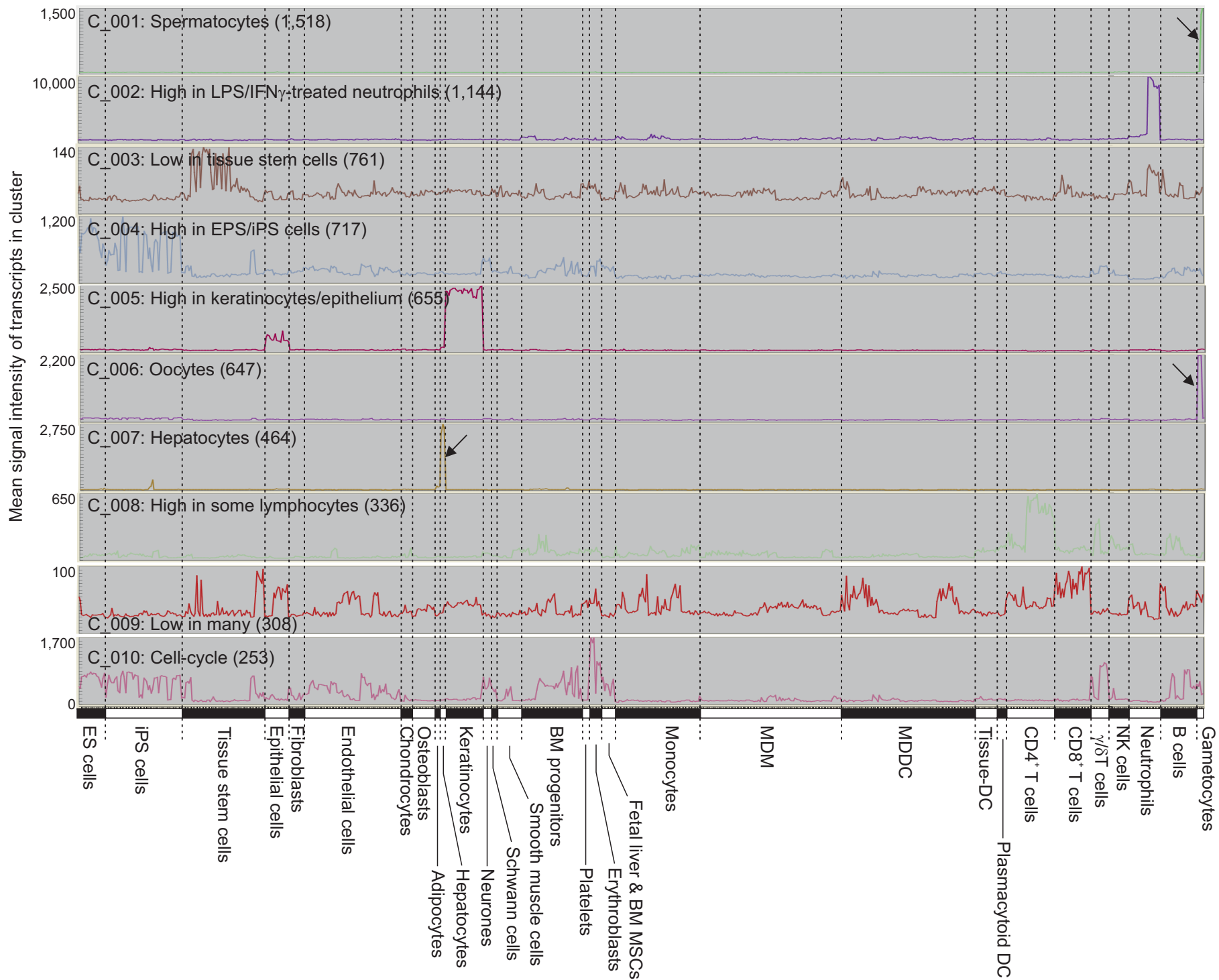

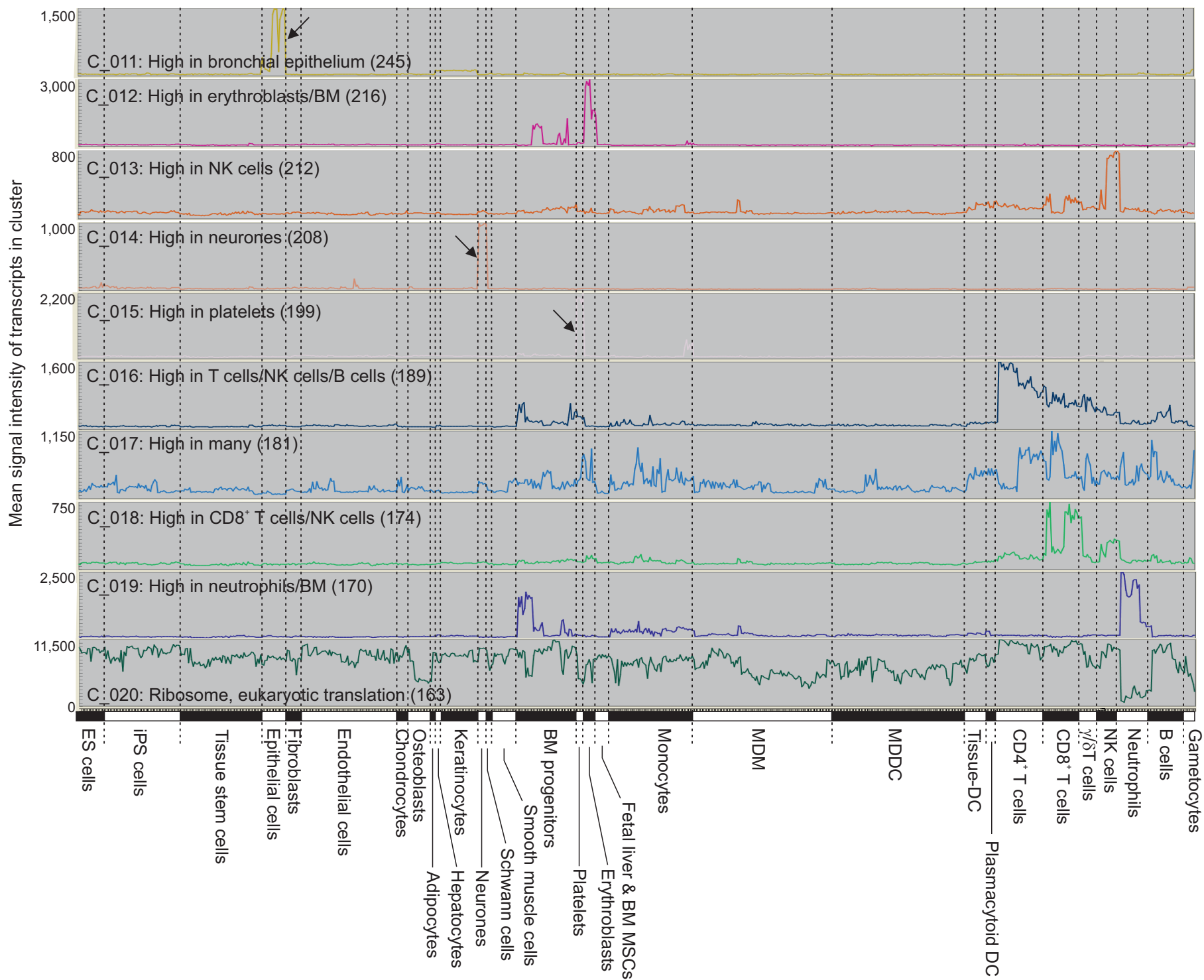

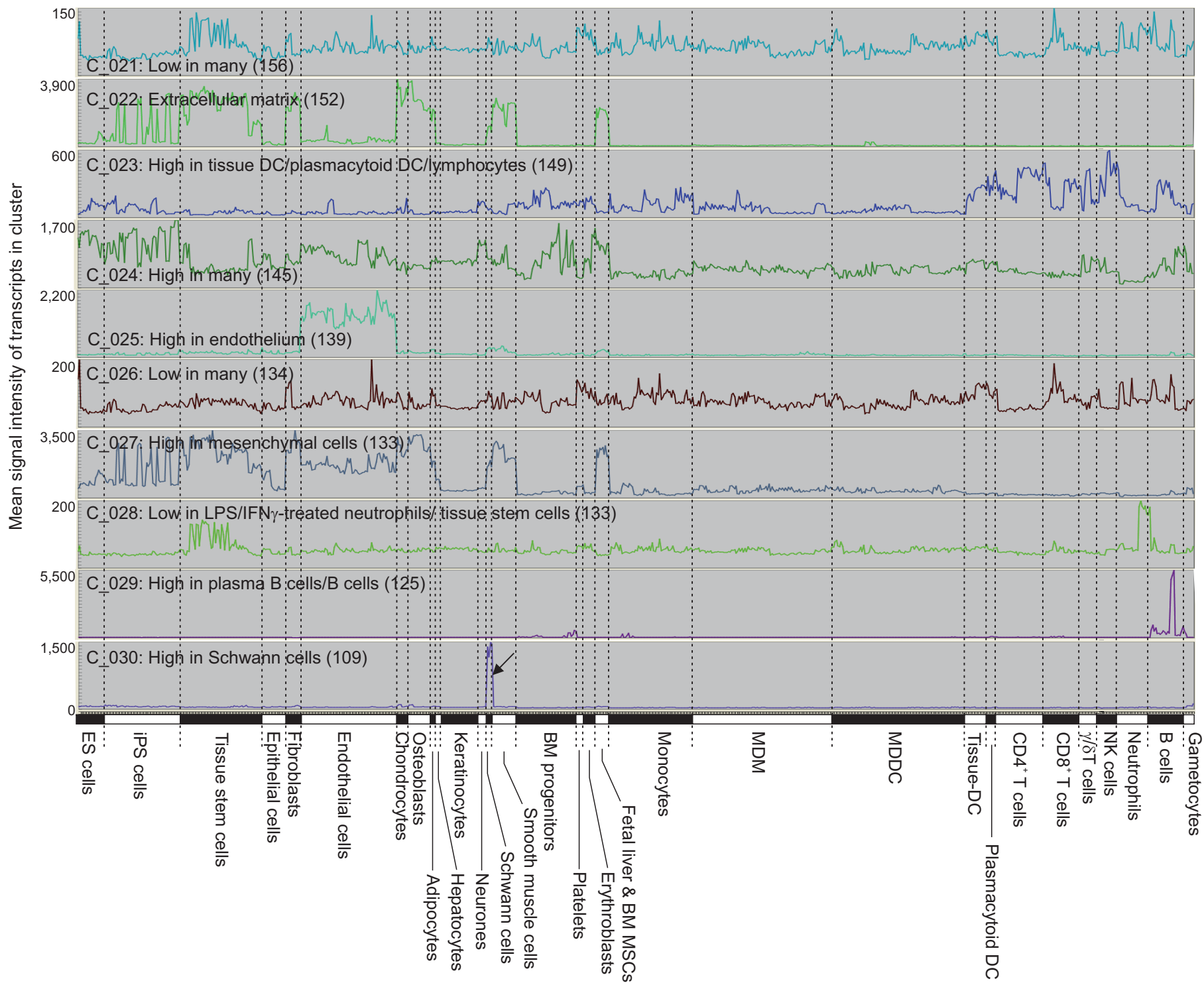

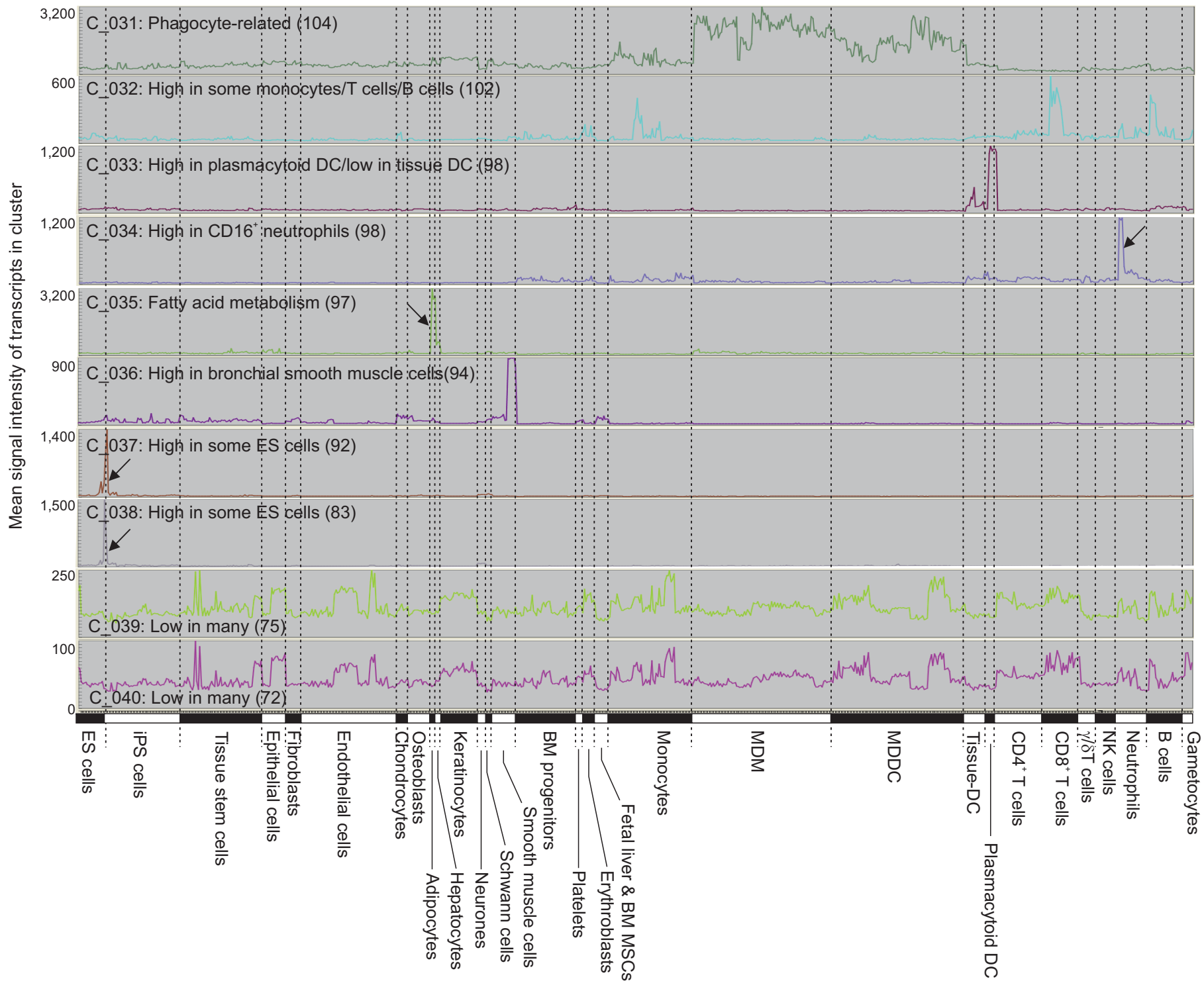

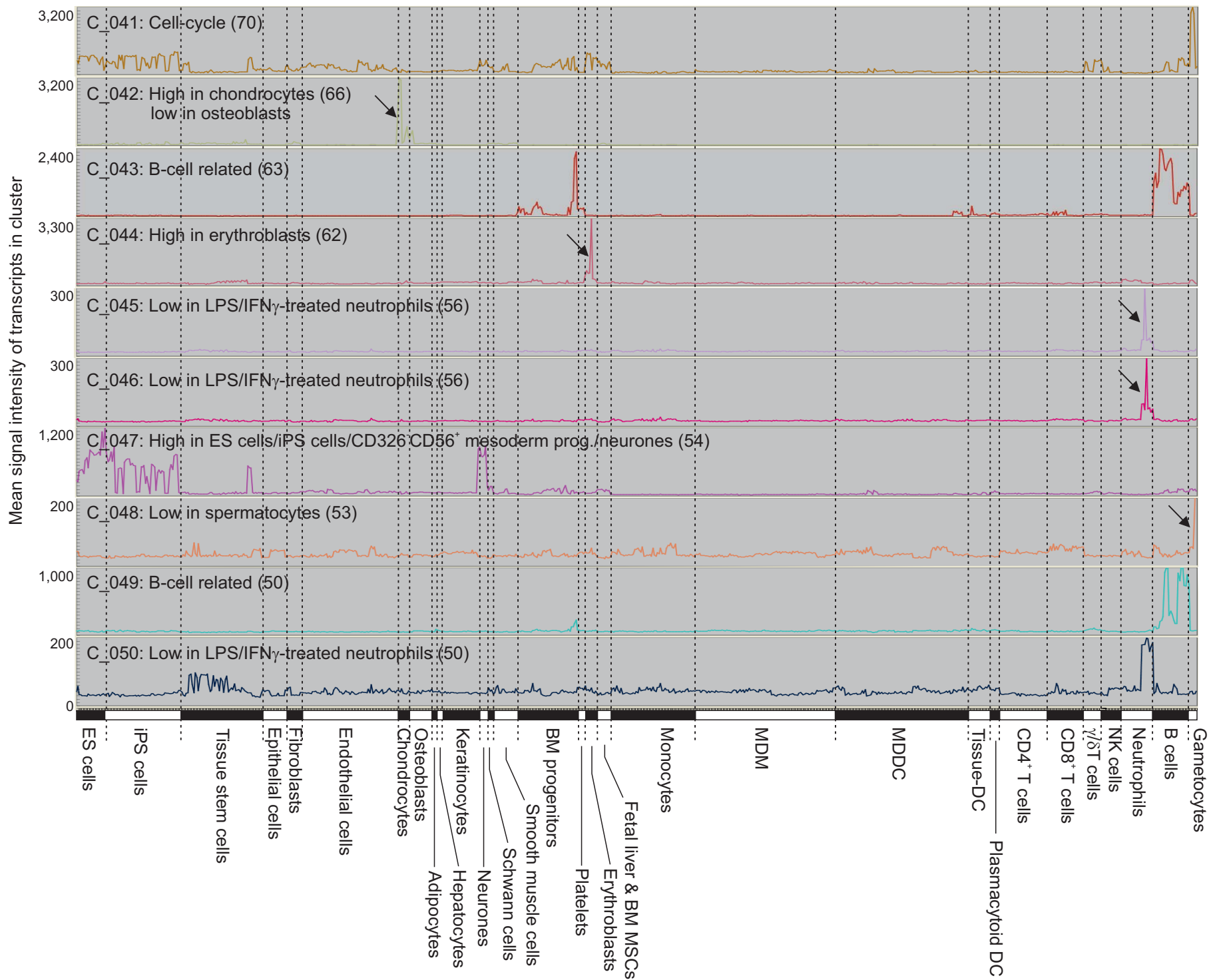

Supplement: Additional file 4: Figure S2 — Average expression profile of all transcripts present in the 50 largest clusters from these analyses. x-axis displays the grouping of the different primary 590 cells analysed and the y-axis the average normalised expression signal of all transcripts in cluster. The cluster number is shown (C_00N), together with the annotated cluster name and the number of transcripts it contains (in brackets). [file 1471-2164-14-632-S4.pdf]

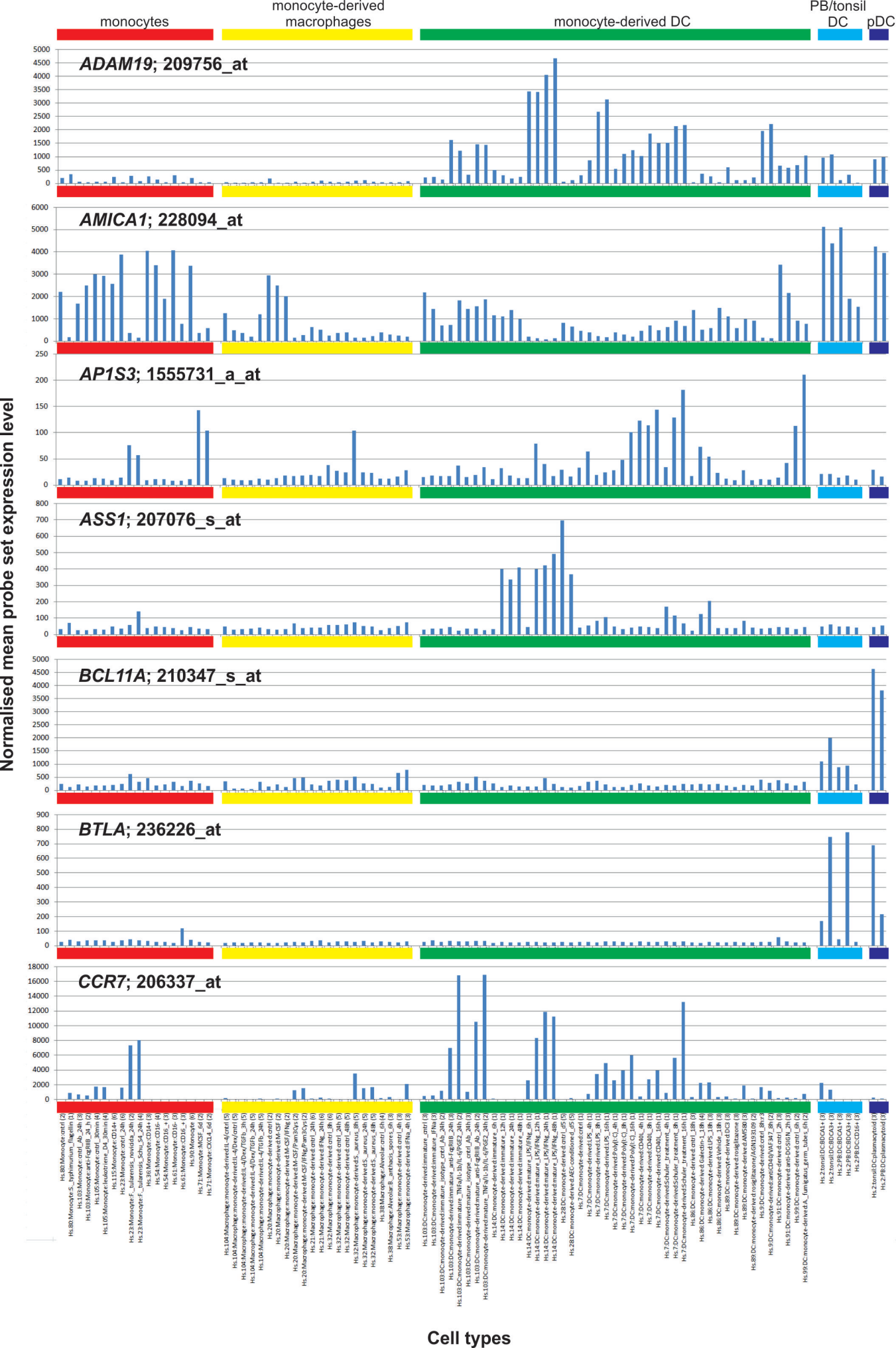

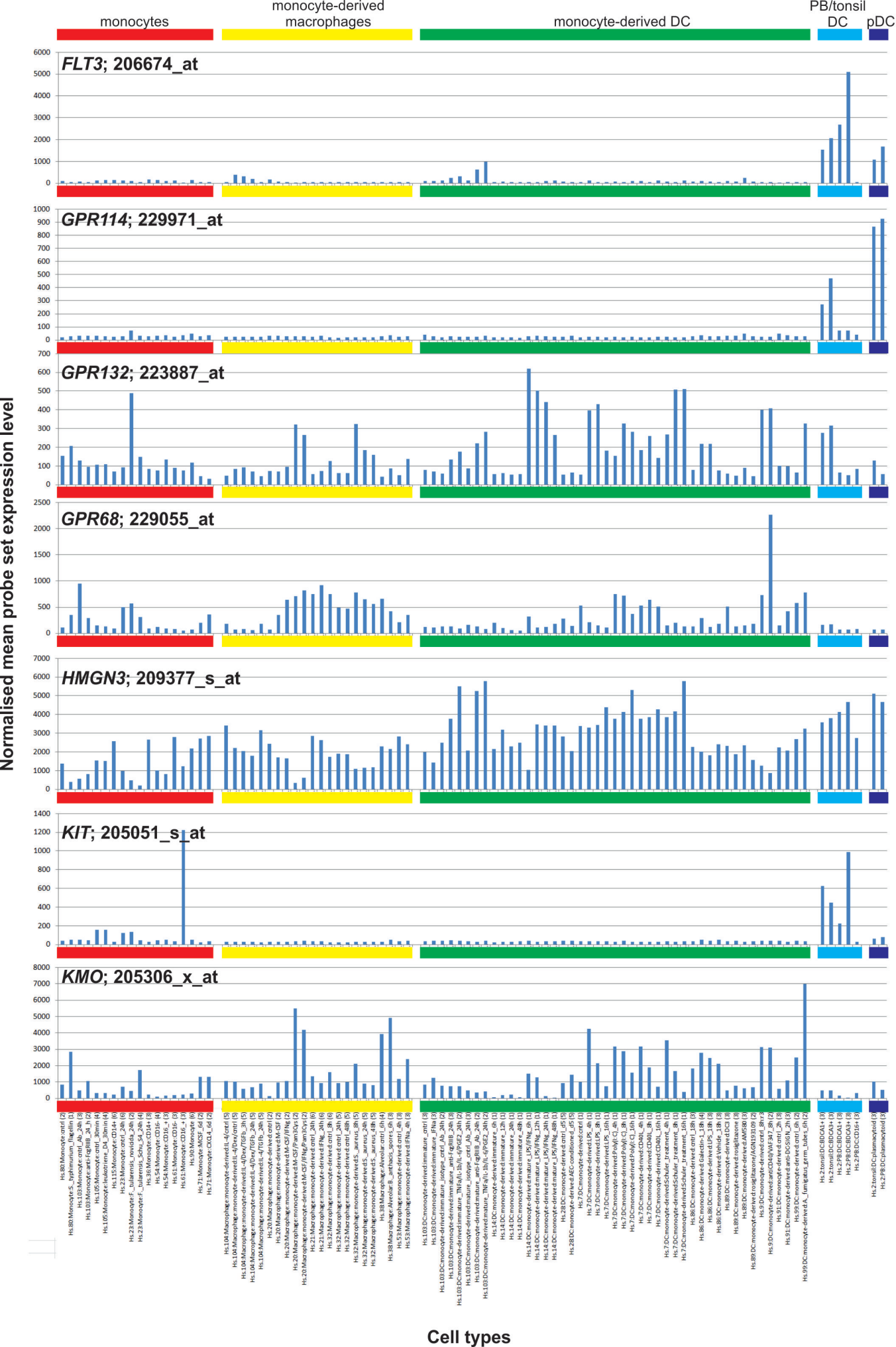

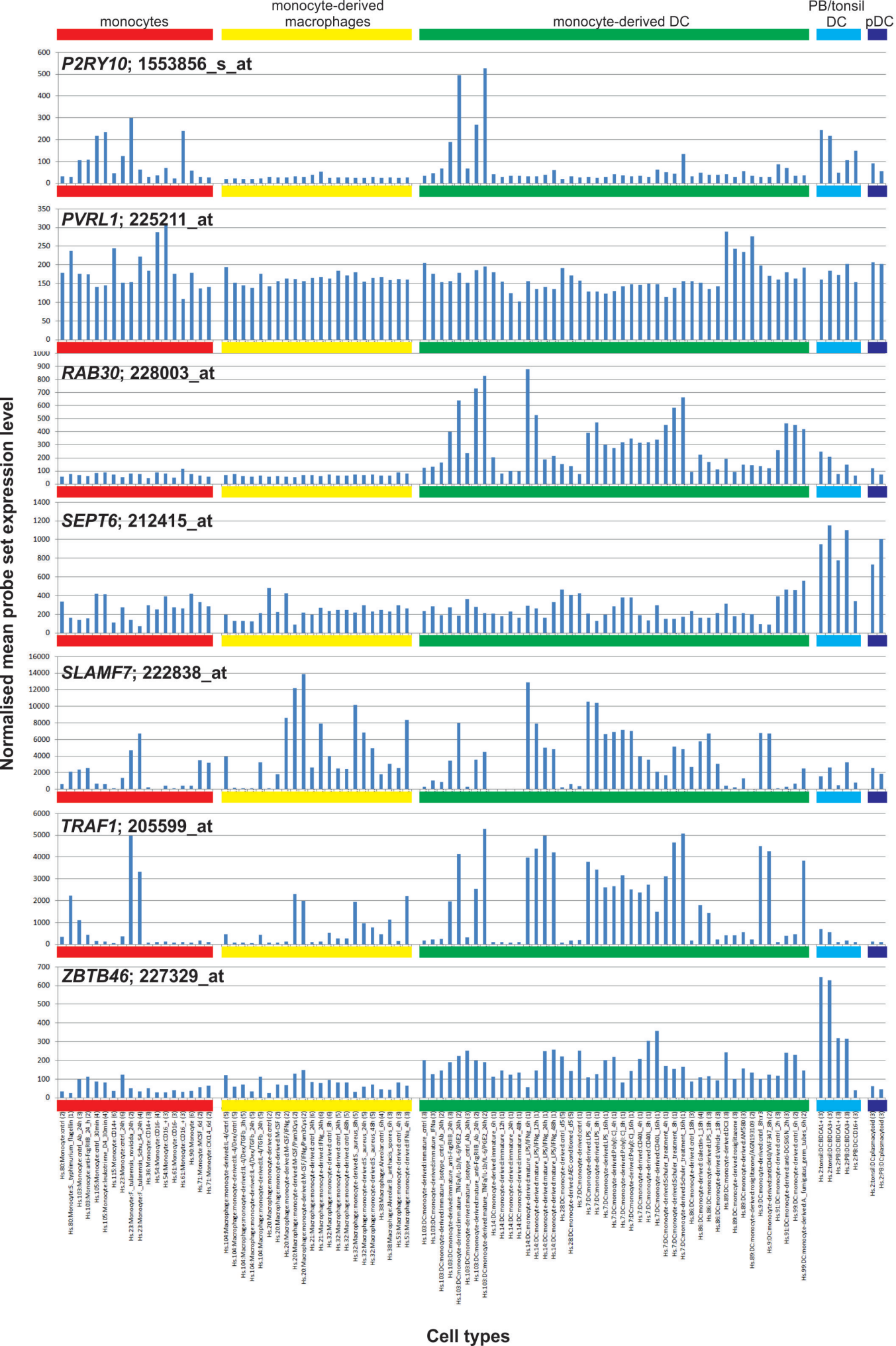

Supplement: Additional file 6: Figure S3 — Expression of mouse dendritic cell ‘marker’ genes across human myeloid cell types analysed in these studies. The list of genes shown here was recently published by Miller et al. (2012) as defining mouse dendritic cells based on the analysis of a subset of the ImmGen data [62,63]. We have suggested that this is not really the case in mice and this figure would suggest that this does not hold true in humans either. Horizontal colour bars represent the myeloid sub-types analysed, the histogram bars the mean 600 expression value for the replicates of an individual sample type from a study, number of samples averaged is shown in brackets. [file 1471-2164-14-632-S6.pdf]
